# Audit and feedback interventions involving pharmacists to influence prescribing behaviour in general practice: a systematic review and meta-analysis

**DOI:** 10.1093/fampra/cmac150

**Published:** 2023-01-12

**Authors:** Mary Carter, Nouf Abutheraa, Noah Ivers, Jeremy Grimshaw, Sarah Chapman, Philip Rogers, Michelle Simeoni, Jesmin Antony, Margaret C Watson

**Affiliations:** Department of Life Sciences, University of Bath, Bath, United Kingdom; School of Medicine, University of Aberdeen, Aberdeen, United Kingdom; Women’s College Research Institute, Women’s College Hospital, Toronto, Ontario, Canada; Centre for Implementation Research, Ottawa Hospital Research Institute, Ottawa, Ontario, Canada; Department of Life Sciences, University of Bath, Bath, United Kingdom; Department of Life Sciences, University of Bath, Bath, United Kingdom; Public Health Ontario, Toronto, Ontario, Canada; Women’s College Research Institute, Women’s College Hospital, Toronto, Ontario, Canada; Strathclyde Institute of Pharmacy and Biomedical Sciences, University of Strathclyde, Glasgow, United Kingdom

**Keywords:** evidence, general practice, pharmacist, prescribing, primary care, systematic review

## Abstract

**Introduction:**

Pharmacists, as experts in medicines, are increasingly employed in general practices and undertake a range of responsibilities. Audit and feedback (A&F) interventions are effective in achieving behaviour change, including prescribing. The extent of pharmacist involvement in A&F interventions to influence prescribing is unknown. This review aimed to assess the effectiveness of A&F interventions involving pharmacists on prescribing in general practice compared with no A&F/usual care and to describe features of A&F interventions and pharmacist characteristics.

**Methods:**

Electronic databases (MEDLINE, EMBASE, CINAHL, Cochrane Central Register of Controlled Trials, (Social) Science Citation Indexes, ISI Web of Science) were searched (2012, 2019, 2020). Cochrane systematic review methods were applied to trial identification, selection, and risk of bias. Results were summarized descriptively and heterogeneity was assessed. A random-effects meta-analysis was conducted where studies were sufficiently homogenous in design and outcome.

**Results:**

Eleven cluster-randomized studies from 9 countries were included. Risk of bias across most domains was low. Interventions focussed on older patients, specific clinical area(s), or specific medications. Meta-analysis of 6 studies showed improved prescribing outcomes (pooled risk ratio: 0.78, 95% confidence interval: 0.64–0.94). Interventions including both verbal and written feedback or computerized decision support for prescribers were more effective. Pharmacists who received study-specific training, provided ongoing support to prescribers or reviewed prescribing for individual patients, contributed to more effective interventions.

**Conclusions:**

A&F interventions involving pharmacists can lead to small improvements in evidence-based prescribing in general practice settings. Future implementation of A&F within general practice should compare different ways of involving pharmacists to determine how to optimize effectiveness.

PRISMA-compliant abstract included in Supplementary Material 1.

**Key messages**

- Audit and feedback (A&F) is effective in changing prescribing behaviour.
- Pharmacist-led A&F influences prescribing in primary care settings.
- Pharmacists in general practice may be ideally situated for delivering A&F.

## Introduction

A growing number of pharmacists are based in general medical practices (also known as *family practices*, *family medicine groups*, or *primary care clinics*), which is the typical point of entry to healthcare systems in many countries, e.g. in Canada, New Zealand, and the Netherlands.^1–3^ The increase in general practice-based pharmacists has been particularly marked in the United Kingdom where their integration is promoted and supported by healthcare policies and professional bodies.^4–7^

Despite extensive guidance to promote evidence-based prescribing, i.e. to optimize the safe, effective, and efficient use of medicines, some unwarranted variation persists.^8,9^ Some variation may be expected, since evidence-based guidelines do not apply in all scenarios, but previous studies have found that some differences are clinically unjustified and associated with disparities in patient outcomes,^10^ medicines waste,^11^ and rising costs.^12^ There is a need to identify and explore the features of strategies that can most effectively encourage health professionals to align their practice with evidence.^13–15^ Pharmacists are adopting various roles which impact prescribing in a range of healthcare settings, including the delivery of audit and feedback (A&F) interventions.^16–21^ An examination of pharmacists’ involvement in the delivery of a proven method for behaviour change (A&F) may contribute to identifying a role in which pharmacists can fully use and develop their expertise.

A&F interventions seek to influence clinical practice through monitoring and reinforcement of positive behaviours.^22^ Specifically, data about individual or group practice are collected and compared with a standard, e.g. evidence-based guidelines, professional standards, or peer performance. This information is fed back to the individual/group to encourage change in practice or closer compliance with the standard.^23^ A 2012 Cochrane review^24^ demonstrated A&F interventions to be effective in achieving health professional behaviour change when feedback is provided by a supervisor or colleague; more than once; both verbally and in writing; and includes clear targets and an action plan. Additional characteristics associated with effective A&F include the credibility of the data used in A&F interventions, opportunity for recipients to discuss feedback, and choice of comparator.^25,26^

This systematic review builds on and forms a discrete part of an ongoing update of the earlier Cochrane review.^24^ It focussed on the effectiveness of A&F interventions involving pharmacists as key contributors on prescribing in general practice.

The specific objectives of the pharmacist-related review were to:

1. Compare the effectiveness of A&F interventions involving pharmacists on prescribing in general practice with usual care or non-A&F interventions.
2. Identify and describe the:
  - features of A&F interventions involving pharmacists
  - characteristics of the pharmacists contributing to A&F interventions

## Methods

The review protocol was registered with the International Prospective Register of Systematic Reviews (PROSPERO), registration number CRD42020194355. This report is guided by the Preferred Reporting Items for Systematic Review and Meta-Analysis (PRISMA) checklist^27^ (Supplementary Material 2).

### Scope of the review

Randomized studies, including cluster and step wedge trials, in general practice (or facilities in which general practitioners [GPs] provided medical services) and which met the following eligibility criteria were included:

**Participants** included were pharmacists involved as sole contributor or part of a team conducting A&F interventions (or similar auditing and feedback techniques) or healthcare professionals who were participants in these interventions or other personnel who were recipients of prescribing feedback on behalf of healthcare professionals. **Interventions** were A&F to influence prescribing, including interventions where A&F (or similar auditing and feedback techniques) was used as a sole method or in combination with other quality improvement techniques. **Comparators** were usual care or non-A&F interventions. **Outcomes** were objectively measured prescribing or healthcare outcomes.

### Information sources

The A&F Systematic Review (A&F SR) Group (see Acknowledgements for membership) conducted searches (without language restrictions): Cochrane Library, clinical trials.gov, MEDLINE (Ovid), EMBASE (Ovid), CINAHL (Ebsco) (from June 2010 to June 2020), and WHO International Clinical Trials Registry (June 2010 to February 2019) to identify studies of A&F interventions (pharmacist and non-pharmacist)^28^ for inclusion in the Cochrane update. Studies from before 2010 were identified from the original Cochrane A&F systematic review.^24^ Details of searches are included in Supplementary Material 3.

Duplicate, independent screening was undertaken (MC, MCW) in May 2020 of all titles and abstracts identified for inclusion in the Cochrane review update by the A&F SR Group, to identify trials that evaluated A&F interventions focussed on prescribing in general practice settings. Reference lists of trials identified for the pharmacist sub-review were searched for additional studies. MC undertook screening of additional trials identified by the 2020 search for inclusion in the Cochrane update in February 2022.

### Data extraction and management

Duplicate data extraction was undertaken for all studies included in the Cochrane update^28^ by members of the A&F SR Group, using the Cochrane Effective Practice and Organization of Care (EPOC) extraction form. Independent, duplicate extraction was undertaken (MC, NA) of additional data items for the pharmacist sub-review, including the number of pharmacists and their role(s) in the intervention, details of the prescribing topic addressed in intervention, pharmacists’ years of experience, and their work situation in relation to participating GPs. Authors of studies for which results data were missing were contacted by email. Data items extracted for the sub-review were added to details concerning study and intervention characteristics extracted for the Cochrane update.

### Risk of bias in individual studies

Duplicate, independent evaluation of the risk of bias was undertaken by members of the A&F SR Group and/or MC and NA, using EPOC-recommended risk of bias methods (adapted from the general Cochrane tool^29^).

Discrepancies between reviewers relating to screening, data extraction, and risk of bias assessment were resolved by exchange of emails and online discussions where further explanations were necessary.

### Summary measures

Where possible, risk ratios (RRs) of appropriate prescribing were calculated using a 95% confidence interval (CI). For other continuous outcomes and where data were available, standardized mean differences and standard deviation were calculated.

### Data synthesis and meta-analysis

All studies were included in the descriptive analysis. Details about the A&F interventions, including the characteristics of the pharmacist(s) involved, were summarized descriptively and frequencies produced. Only studies deemed sufficiently homogenous in design and outcome were included in a meta-analysis.^30^ Included outcomes concerned potentially inappropriate or risky prescribing, or prescribing that did not comply with specified guidelines. Cochrane Review Manager (RevMan) v5.4 software was used to produce a random-effects model. Effect sizes were calculated using the Mantel–Haenszel RR and 95% CIs. Heterogeneity was assessed using the *I*^2^ statistic. A funnel plot for assessment of bias across studies was not considered appropriate, due to the low number of studies included in the meta-analysis.^30^

## Results

**Fig. 1.**
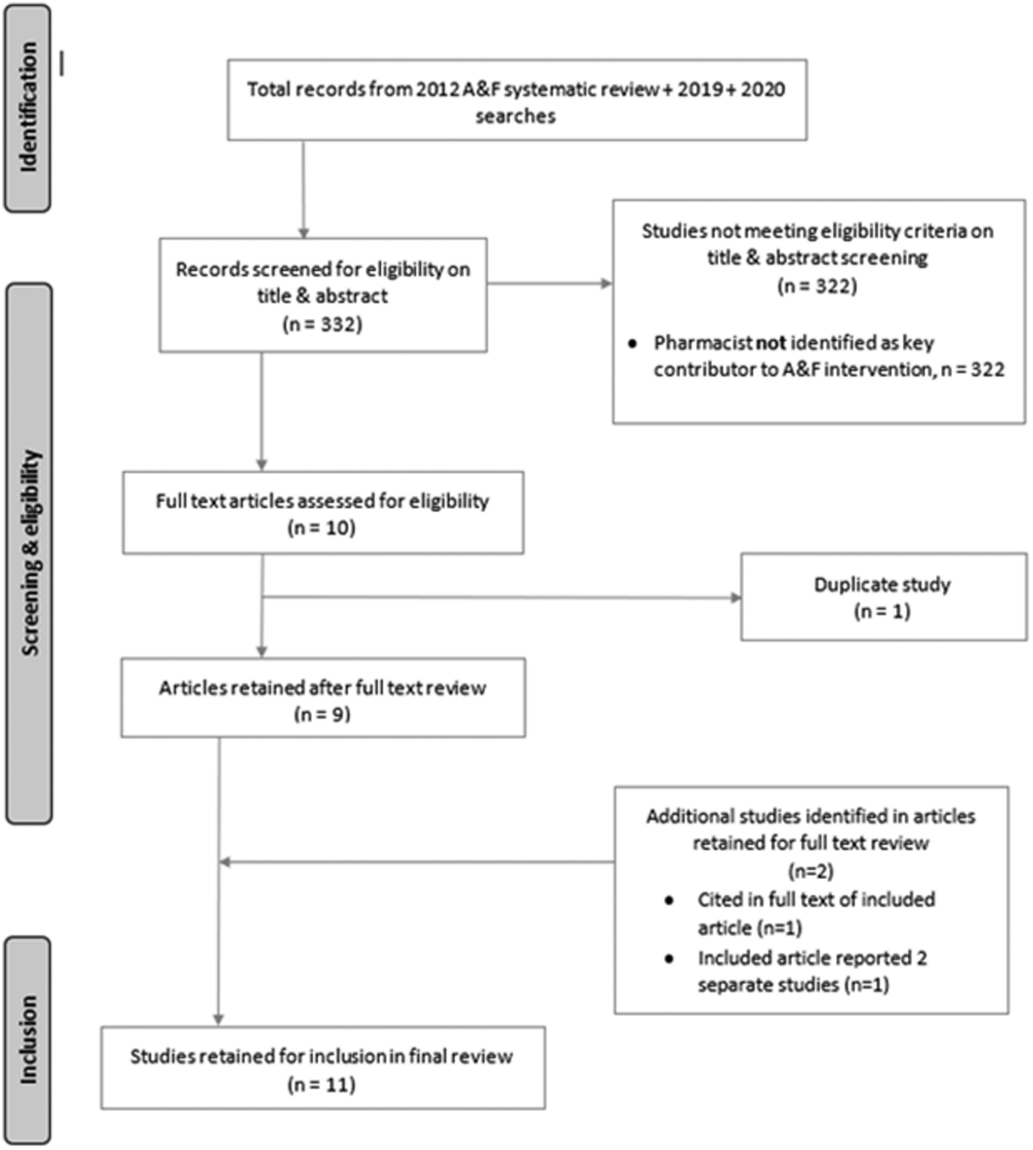
PRISMA flow diagram of A&F intervention studies identified and screened for inclusion in the final review.

Of the 332 studies identified for inclusion in the Cochrane update,^28^ 11 were included in this pharmacist-focussed review (Fig. 1). The studies were conducted in 9 countries: 2 each from the Netherlands^31,32^ and Italy^33^ and one each from the United Kingdom,^34^ Denmark,^35^ Norway,^36^ Republic of Ireland,^37^ Australia,^38^ United States,^39^ and Malaysia.^40^ The article from Italy reported 2 studies,^33^ and these were treated as 2 separate studies for the purpose of this review.

The percent agreement between raters (screening, data extraction, and risk of bias assessment) was 84%.

### Characteristics of included studies

Nine studies included 2 arms (intervention, control) (Table 1). Two 3-armed studies^35,40^ were included with full intervention, partial intervention, and control arms. The median number of participating practices/clinics was 47 (range 8^32^ to 146^36^), with 279 clinicians (range 41^35^ to 1,737^33^) and 1,884 patients (range 196^37^ to 63,337^34^).

**Table 1.** Characteristics of studies, including description of participants and intervention.

Study (first author + date of publication)	Objective	Country	Date of intervention	Unit of randomization (no. randomized)	Participants & setting	Intervention	Control
Lim, 2018^40^	To reduce prescribing errors	Malaysia	May to December 2012	Health district (10) **Full intervention:** 4 health districts 24 clinics 154 prescribers **Partial intervention:** 3 health districts 14 clinics 105 prescribers **Control** 3 health districts 13 clinics 92 prescribers	Prescribers (family medicine specialists, medical officers, medical assistants) in government primary care clinics Number of pharmacists not reported	**Full intervention** Prescription review by pharmacist League tables Authorized feedback letter **Partial intervention** Prescription review by pharmacist League tables **Duration:** review & feedback for 3 months	Prescription review by pharmacist
Trietsch, 2017^31^ (a)	To reduce inappropriate testing & prescribing in 5 clinical areas	The Netherlands	January 2008 to December 2010	Local Quality Improvement Collaborative (LQIC) (21) **Intervention** 10 LQICs 39 practices 86 GPs **Control** 11 LQICs 49 practices 122 GPs	GPs participating in LQICs 39 pharmacists	Group discussion & individual feedback in LQIC (moderated by pharmacist) about tests ordered & drugs prescribed in clinical area a: • Anaemia • Dyslipidemia • Prostate complaints • Rheumatic complaints • Urinary tract infection **Duration:** 3 meetings lasting 90–120 min	Group discussion & individual feedback in LQIC about tests ordered & drugs prescribed in clinical area b: • Chlamydia trachomatis • Diabetes type 2 • Stomach complaints • Perimenopausal complaints • Thyroid dysfunction
Trietsch, 2017^31^ (b)	To reduce inappropriate testing & prescribing in 5 clinical areas	The Netherlands	January 2008 to December 2010	**Intervention** 11 LQICs 49 practices 122 GPs **Control** 10 LQICs 39 practices 86 GPs	GPs participating in LQICs 49 pharmacists	Group discussion & individual feedback in LQIC (moderated by pharmacist) about tests ordered & drugs prescribed in clinical area b: • Chlamydia trachomatis • Diabetes type 2 • Stomach complaints • Perimenopausal complaints • Thyroid dysfunction **Duration:** 3 meetings lasting 90–120 min	Group discussion & individual feedback in LQIC about tests ordered & drugs prescribed in clinical area a: • Anaemia • Dyslipidemia • Prostate complaints • Rheumatic complaints • Urinary tract infection
Vervloet, 2016^32^	To reduce antibiotic prescribing for respiratory tract infections	The Netherlands	2010–2012	Pharmacotherapy Audit Meeting (PTAM) (8) **Intervention** 4 PTAMs 39 FPs **Control** 4 PTAMs 38 FPs	Family physicians (FPs) participating in PTAMs Team of pharmacists	Group discussion in PTAMs (FPs & pharmacists) Communication skills training Prompts to guide prescribing in electronic prescribing systems Feedback of prescribing data **Duration:** unclear	No intervention
Clyne, 2015^37^	To reduce inappropriate prescribing for older patients	Republic of Ireland	October 2012 to September 2013	General practice (21) **Intervention** 11 general practices 99 patients **Control** 10 general practices 97 patients	GPs in general practices Number of pharmacists not reported	Academic detailing visit from pharmacist incorporating medicines review & web-based treatment recommendations **Duration:** 1 meeting lasting 30 min	Usual care & simple patient-level feedback
Magrini (TEA), 2014^33^ (a)	To increase appropriate prescribing for osteoporosis	Italy	Spring 2007 to Winter 2007/2008	Primary Care Group (PCG) (115) **Intervention** 57 PCGs 853 GPs **Control** 58 PCGs 884 GPs	GPs participating in PCGs Number of pharmacists not reported	Group discussion/continuing medical education (CME) with pharmacists about a “therapeutic area” approach to prescribing Provision of information about osteoporosis **Duration:** 2 meetings lasting 3–4 h	Group discussion/CME with pharmacists about a “therapeutic area” approach to prescribing Provision of information about prostatic hyperplasia
Magrini (TEA), 2014^33^ (b)	To increase appropriate prescribing for prostatic hyperplasia	Italy	Spring 2007 to Winter 2007/2008	PCG (115) **Intervention** 58 PCGs 884 GPs **Control** 57 PCGs 853 GPs	GPs participating in PCGs Number of pharmacists not reported	Group discussion/CME with pharmacists about a “therapeutic area” approach to prescribing Provision of information about prostatic hyperplasia **Duration:** 2 meetings lasting 3–4 h	Group discussion/CME with pharmacists about a “therapeutic area” approach to prescribing Provision of information about osteoporosis
Magrini (SIDRO), 2014^33^ (a)	To reduce prescribing of barnidipine (antihypertensive)	Italy	Spring 2007 to Winter 2007/2008	PCG (115) **Intervention** 57 PCGs 843 GPs **Control** 58 PCGs 892 GPs	GPs participating in PCGs Number of pharmacists not reported	Group discussion/CME with pharmacists about a “single drug oriented” approach to prescribing Provision of information about barnidipine, including drug utilization data & clinical scenarios **Duration:** 2 meetings lasting 3–4 h	Group discussion/CME with pharmacists about a “single drug oriented” approach to prescribing Provision of information about prulifloxacin, including drug utilization data & clinical scenarios
Magrini (SIDRO), 2014^33^ (b)	To reduce prescribing of prulifloxacin (antibiotic)	Italy	Spring 2007 to Winter 2007/2008	PCG (115) **Intervention** 58 PCGs 892 GPs **Control** 57 PCGs 843 GPs	GPs participating in PCGs Number of pharmacists not reported	Group discussion/CME with pharmacists about a “single drug oriented” approach to prescribing Provision of information about prulifloxacin, including drug utilization data & clinical scenarios **Duration:** 2 meetings lasting 3–4 h	Group discussion/CME with pharmacists about a “single drug oriented” approach to prescribing Provision of information about barnidipine, including drug utilization data & clinical scenarios
Avery, 2012^34^	To reduce unsafe prescribing & inadequate monitoring in selected areas of medicines management	England, United Kingdom	January 2006 to January 2009	General practice (72) **Intervention** 36 general practices 32,938 patients **Control** 36 general practices 30,399 patients	GPs in general practices 72 pharmacists	Pharmacist-led feedback & educational outreach Computerized decision support Dedicated support from pharmacists to GPs **Duration:** 12 weeks of feedback & support	Computer-generated simple feedback for at-risk patients
Pape, 2011^39^	To improve care (including prescribing) for patients with diabetes mellitus	United States	Unclear	Primary care clinic (PCC) (9) **Intervention** 3 PCCs 2,069 patients **Control** 6 PCCs 4,160 patients	Physicians in PCCs Number of pharmacists not reported	Access & training on software providing reporting, benchmarking & decision support Implementation of physician/pharmacist team-based care for diabetes patients **Duration:** 24 months of team-based care (pharmacist & physician)	Access & training on software providing reporting, benchmarking & decision support
Bregnhoj, 2009^35^	To reduce inappropriate prescribing for older patients	Denmark	Prior to June 2007	GP (41) **Combined intervention** 15 GPs 79 patients **Single intervention** 12 GPs 61 patients **Control** 14 GPs 72 patients	GPs in general practices 1 pharmacist	**Combined intervention** Interactive educational meeting Prescribing recommendations and feedback from pharmacists for specific patients **Single intervention** Interactive educational meeting **Duration:** unclear	No intervention
Fretheim, 2006^36^	To increase evidence-based prescribing of antihypertensives & cholesterol-lowering drugs	Norway	May to December 2002	General practice (146) **Intervention** 73 general practices 1,626 patients **Control** 73 general practices 1,426 patients	GPs in general practices 4 pharmacists	Educational outreach visit from pharmacist, including presentation of evidence from guidelines, choice of first-line drugs & treatment goals. Computerized reminders & prescribing recommendations to GPs **Duration:** unclear	Guidelines sent to practices
Crotty, 2004^38^	To increase evidence-based clinical care (including prescribing) for falls reduction & stroke prevention	Australia	Unclear	Residential facility (20) **Intervention** 10 residential facilities 381 patients **Control** 10 residential facilities 334 patients	GPs caring for patients in residential facilities Number of pharmacists not reported	Two 30-min academic detailing visits by pharmacist, including presentation of relevant evidence (guidelines), information from case note audit & facility’s falls rates, prescribing patterns & risk reduction practices. Trained link nurse in facility. Visit by pharmacist visit to encourage reduction in psychotropic medications prescription. Surveys of staff & GPs **Duration:** 2 visits lasting 30 min	Case notes audit Surveys of staff & GPs

In 3 studies, control group participants received no active intervention^32,35,36^; in 1 study, control group participants had access to the same prescribing and benchmarking data as intervention group participants but did not implement a team-based care system to optimize this knowledge.^39^ In all other studies, control group participants received a non-A&F intervention such as access to information technology resources or guidelines, or prescription review only.

GPs were the recipients of the A&F intervention in all studies. The interventions took place in general practices or primary care clinics in all studies apart from one which focussed on GPs’ care for patients in residential care facilities.^38^

All A&F interventions included outcomes associated with prescribing (Table 2). The median number of prescribing outcomes was 2 (range 1^32,35,36,39,40^ to 19^31^). Eight studies included outcomes which aimed to reduce prescribing errors or inappropriate prescribing. In the 3 other studies, the outcome was an increase in a desired prescription of selected medications for osteoporosis and prostatic hyperplasia,^33^ thiazide for hypertension,^36^ and lipid-lowering medication.^39^

**Table 2.** Effects of A&F interventions on prescribing.

Study	Outcome measure (Total number of prescribing outcomes reported)	Intended direction of change	Effect of intervention	Follow-up (& losses to follow-up—LTF)
Lim, 2018^40^	Prescriptions with errors (1)	↓	Tx*: 2,641/7,280 prescriptions (36.3%) Cx: 2,102/3,920 prescriptions (53.6%) RR: 0.68 (0.65–0.71)	4 months Tx: No clinics LTF; Cx: No clinics LTF
Trietsch, 2017^31^	Mean no. of DDD antibiotic prescriptions for UTI/6 months/1,000 patients (19)	↓	Tx: 47.3 (36.5)/86 GPs Cx: 59.7 (48.7)/122 GPs SMD: −0.28 (−0.56, −0.00)	9 months Tx topic group A (Cx topic group B): 1 LQIC (10 GPs) LTF Cx topic group A (Tx topic group B): 2 LQICs (17 GPs) LTF
Vervloet 2016^32^	Mean no. antibiotic prescriptions for RTI/year/1,000 patients (1)	↓	Tx: 155 (51.7)/59,483 patients Cx: 160 (35.8)/94,767 patients SMD −0.11 (−0.12, −0.10)	12 months Tx: None LTF; Cx: None LTF
Clyne, 2015^37^	Potentially inappropriate prescriptions (12)	↓	Tx: 52/96 patients (52.5%) Cx: 75/94 patients (77.3%) RR: 0.68 (0.55–0.84)	5 months Tx: 3 patients LTF; Cx: 3 patients LTF
Magrini (TEA), 2014^33^	Appropriate prescriptions for osteoporosis or prostatic hyperplasia (4)	↑	Results data not available	6 months Tx therapeutic area A (Cx therapeutic area B): 1 PCG (56 GPs) LTF Cx therapeutic area A (Tx therapeutic area B): 76 GPs LTF
Magrini (SIDRO), 2014^33^	Prescriptions for barnidipine or prulifloxacin (2)	↓	Results data not available	6 months Tx drug A (Cx drug B): 3 PCGs (92 GPs) LTF Cx drug A (Tx drug B): 79 GPs LTF
Avery, 2012^34^	At least 1 prescription problem/at risk of at least 1 prescription problem (11)	↓	Tx: 553/24,073 patients (2.3%) Cx: 752/26,329 patients (2.9%) RR: 0.80 (0.72–0.90)	6 months Tx: No general practices LTF; Cx: No general practices LTF
Pape, 2011^39^	Prescriptions for lipid-lowering medication (1)	↑	Tx: 471/2,047 patients (23.0%) Cx: 1,819/4,916 patients (37.0%) RR: 0.62 (0.57–0.68)	24 months Tx: No primary care clinic LTF; Cx: No primary care clinic TF
Bregnhoj, 2009^35^	Medications Appropriate Index score (1)	↓	Tx*: 6/49 GPs Cx: 10.1/64 GPs Insufficient data for SMD calculation	12 months Tx: 8 patients LTF; Cx: 8 patients LTF
Fretheim, 2006^36^	Prescriptions for thiazide (1)	↑	Tx: 706/854 patients (83.0%) Cx: 683/768 patients (89.0%) RR: 0.93 (0.89–0.97)	12 months Tx: No general practices LTF; Cx: No general practices LTF
Crotty, 2004^38^	Prescriptions for any psychotropic medication (3)	↓	Tx: 266/381 patients (69.9%) Cx: 227/334 patients (68.0%) RR: 1.03 (0.93–1.13)	7 months Tx: No residential facilities LTF; Cx: No residential facilities LTF

*3-arm study—results shown for 2 arms only (full A&F intervention vs. control). DDD, defined daily dose; LTF, lost to follow-up; LQIC, Local Quality Improvement Collaborative; SMD, standardized mean difference; Tx, treatment (intervention); Cx, control; UTI, urinary tract infection; RTI, respiratory tract infection.

The implementation of a guideline for the use of antihypertensive and cholesterol-lowering drugs was used as a specific target for participants in 1 study.^36^ Clinical and prescribing guidelines were explicitly mentioned in descriptions of interventions, e.g. as the basis for discussions and education sessions, in 6 studies.^31,32,34,35,38,40^ These included guidelines used internationally, e.g. World Health Organization^41^ and British National Formulary^42^ and national guidelines, e.g. Dutch College of GPs (NHG)^43^ and Norwegian General Practice.^44^ Two studies (reported together)^33^ explicitly stated that clinical guidelines were not selected as a comparator because they were viewed with suspicion by participating clinicians.

In 4 studies^31,33,35^ prescribing data were sourced from regional or local databases and in 3 studies the research team extracted computerized data from the practice clinical system.^32,34,36^ For the remaining studies, data from manual charts or prescriptions were used.^37–40^ An association between the source of the data and the effect of the A&F intervention was not observed.

### Risk of bias

**Fig. 2.**
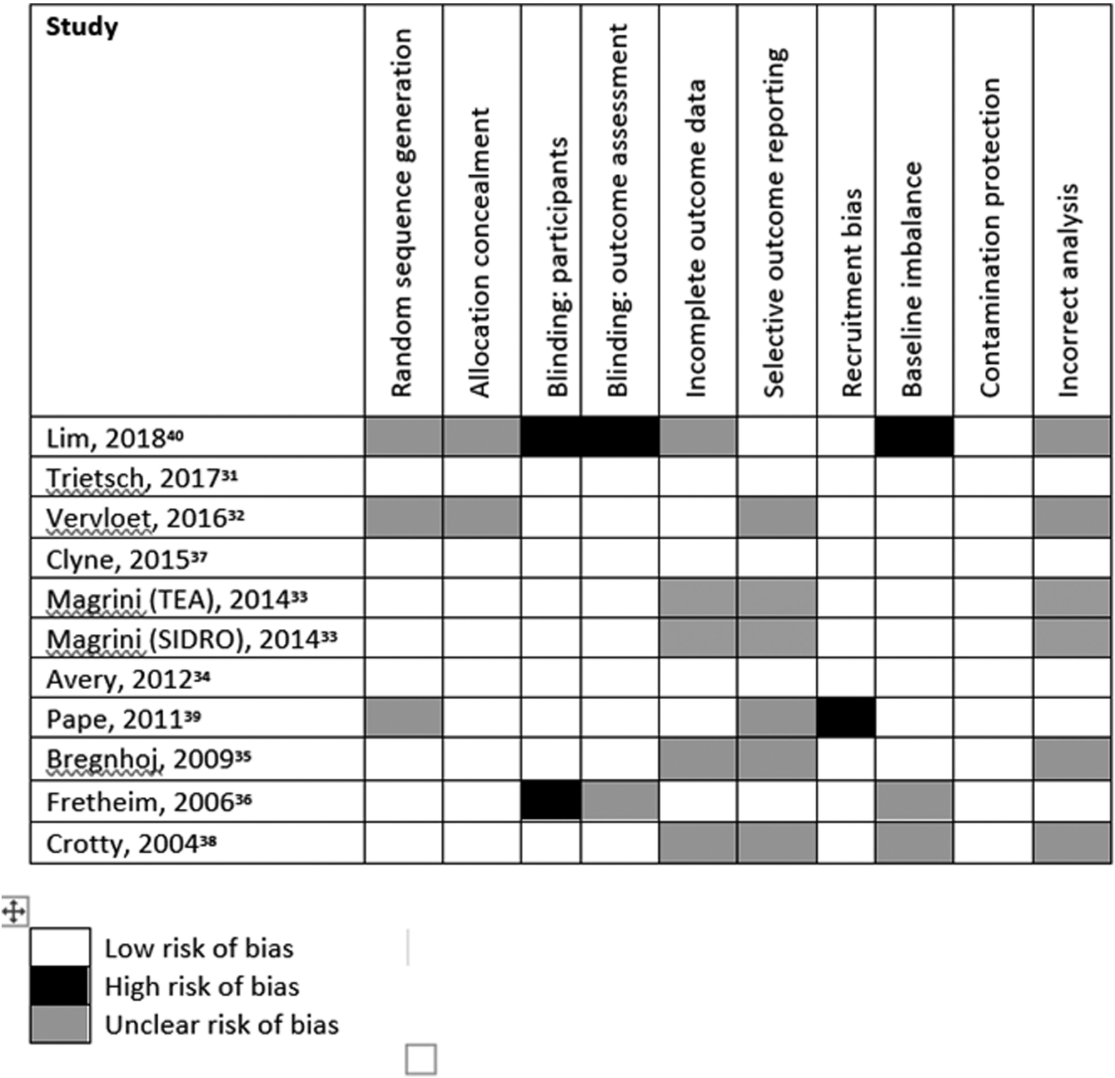
Risk of bias in included studies.

Three studies were assigned low risk of bias for all 10 domains evaluated^31,34,37^ and a further 5 scored low risk for 7 of the domains^33,35,36,39^ (Fig. 2). *Blinding of participants and personnel* were assigned high risk in 2 studies,^36,40^ while in 2 other studies,^32,40^ both *random sequence generation* and *allocation concealment* were assessed as unclear. Both *selective outcome reporting* and *incorrect analysis* were assessed as unclear in 6 studies each (^32,33,35,38,39^ and ^32,33,35,38,40^, respectively).

### Effectiveness of pharmacist A&F intervention

**Fig. 3.**
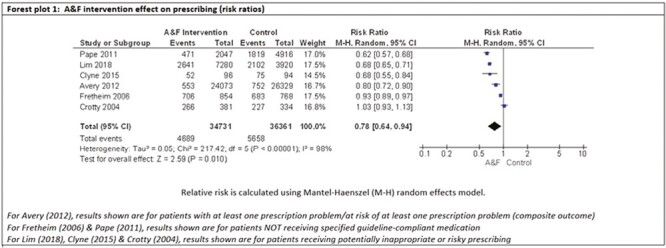
Forest plot of intervention effect sizes.

Six studies (*N* = 71,092) were included in a meta-analysis (Fig. 3). The purpose of 4 of these studies was to reduce inappropriate prescribing^34,37,38,40^ and to increase guideline-compliant prescribing in the 2 remaining studies.^36,39^

The pooled RR across these 6 studies was 0.78 (95% CI: 0.64–0.94), demonstrating that the risk of inappropriate/non-compliant prescribing was 22% lower following an A&F intervention than after usual care or control conditions. High levels of heterogeneity were detected (*I*^2^ = 98%). A funnel plot was not constructed to assess bias due to the small number of studies included in the meta-analysis.^45^

The 5 studies not represented in the meta-analysis had a range of different outcome measures including: the number of antibiotic prescriptions for urinary tract infection^31^ and respiratory tract infection^32^; and a Medication Appropriate Index^35^ score. Two of the studies excluded from the meta-analysis showed improved prescribing in the intervention group^32,35^ but this was not demonstrated in a third study.^31^ No numerical results were available for the remaining 2 studies (reported in 1 paper).^33^

### Determinants of A&F effectiveness

The following results are organized under 3 headings which reflect groups of factors which have been identified as determinants of A&F effectiveness^24–26^: (i) A&F intervention process, (ii) content of feedback reports, (iii) characteristics of the individual (pharmacist) delivering the A&F intervention (Table 3).

**Table 3.** Details of A&F intervention and pharmacist characteristics.

	Lim, 2018	Trietsch, 2017	Vervloet, 2016	Clyne, 2015	Magrini (TEA), 2014	Magrini (SIDRO), 2014	Avery, 2012	Pape, 2011	Bregnhoj, 2009	Fretheim, 2006	Crotty, 2004
Comparator used in feedback report											
Score from prior period(s)	✓	✓						✓	✓		✓
Scores from similar peers	✓				✓	✓					
No comparator mentioned			✓	✓			✓			✓	
Source of data											
Manual chart	✓			✓				✓			
Routinely collected data		✓			✓	✓			✓		✓
Computerized search			✓				✓	✓		✓	✓
Pharmacist role in A&F*	AUD, FRP	GPD	GPD, ED	AUD, ED	GPD, ED	GPD, ED	IPT, ED	AUD, IPT, ED	IPT	AUD, FRP, ED	AUD, FRP, ED
Format of feedback											
Verbal	✓	✓	✓	✓	✓	✓	✓	✓	✓	✓	✓
Written	✓	✓			✓	✓	✓	✓	✓		
Mode of giving feedback											
In-person		✓	✓	✓	✓	✓	✓				✓
Telephone								✓	✓		
Post	✓										
Feedback content											
General	✓	✓	✓	✓	✓	✓		✓	✓	✓	✓
Specific		✓		✓			✓	✓	✓		
Frequency of feedback											
Once					✓	✓	✓	✓	✓	✓	✓
More than once	✓		✓								
Feedback recipient											
Individual	✓			✓			✓	✓	✓	✓	✓
Group		✓	✓		✓	✓	✓				
Decision support tool included			✓	✓			✓	✓		✓	
Level of information in feedback											
Team	✓	✓	✓		✓	✓	✓				✓
Clinician	✓	✓	✓						✓	✓	
Patient				✓			✓	✓	✓	✓	✓
Work situation											
External to participants				✓			✓		✓	✓	✓
Colleague of participants	✓							✓			
Interprofessional group		✓	✓		✓	✓					
Study-specific training		✓	✓	✓	✓	✓				✓	
Experience	Varied	“Senior”	7–30	6	NR	NR	0–24	NR	NR	15–25	NR
Number of pharmacists involved	NR	39	“Team”	NR	NR	NR	72	NR	1	4	NR

Pharmacist role in A&F: AUD conducted audit; FRP provided feedback report; GPD facilitated group feedback discussion with GPs; IPT provided feedback on patient-by-patient basis; ED provided education. NR, not reported.

(i) ***A&F intervention process***

The A&F intervention was incorporated into educational sessions led by pharmacists in 5 studies^34–38^; in 4 of these studies appropriate prescribing in the intervention group improved more than in the control group. This included the 2 studies^35,37^ in which the sessions were described as “interactive.”

In 4 further studies, A&F was incorporated into meetings (lasting up to 3 h) of pre-existing collaborative groups of GPs^31,33^ or GPs and pharmacists.^32^ Meetings included pharmacist-facilitated discussions and/or problem-based learning in interprofessional groups. These studies had mixed results.

The 2 remaining studies included skills training for participants^32,39^ and showed more favourable results for prescribing in the intervention groups.

Five studies involved computerized decision support for prescribing,^32,34,36,37,39^ all of which showed increased appropriate prescribing in the intervention group compared with control.

Pharmacists provided ongoing prescribing support (12 weeks to 2 years) for individual patients in 3 studies^34,35,39^ all of which reported increased appropriate prescribing in the intervention group relative to control.

In 1 study^40^ the pharmacist visited participating clinics to collect and screen handwritten prescriptions from participants on a monthly basis. They provided feedback to participants by post for 3 months; results showed increased appropriate prescribing in the intervention group.

Several studies (*n* = 7) included only 1 episode of feedback^33–36,38,39^; in the 2 studies^32,40^ which included 3 episodes of feedback, the A&F intervention had a small effect. The number of episodes of feedback was given was unclear in the 2 remaining studies.^31,37^

(ii) ***Content of feedback reports***

In 2 studies^36,40^ general information about the prescribing topic was included in feedback reports; both studies showed improvements in prescribing. Four studies comprised feedback reports that combined general information about the clinical topic of interest as well as specific plans developed for or with individual participants.^31,35,37,39^ Three of these studies included action plans for individual participants,^35,37,39^ and all achieved positive effects on prescribing in the intervention group.

In 1 study, prescribers in the intervention group received individual plans based upon discussion with research pharmacists, and their prescribing improved compared with control group prescribers who received general information only.^33^

Seven studies included team-level data in their feedback,^31–34,38,40^ individual clinician-level data were fed back in 5 studies,^31,32,35,36,40^ and individual patient-level data were included in 6 studies.^34–39^ Whilst evaluations of feedback of individual clinician-level data showed variable effect, most studies of individual patient-level data had positive effects.^34–37,39^

Feedback was provided in both verbal and written formats in 7 studies,^31,33–35,39,40^ 4 of which achieved more favourable results in the intervention group compared with the control.^34,35,39,40^ Of the 4 studies which evaluated only verbal feedback,^32,36–38^ 3 reported more favourable results in the intervention groups.

(iii) ***Characteristics of the pharmacist delivering the A&F intervention***

The pharmacist was a colleague of participating GPs in 2 studies^39,40^ and external to the practices in the 9 remaining studies. In 4 studies, the pharmacist was known to participants from regular interprofessional meetings.^31^ Whether the pharmacist was internal or external to the general practice did not make a substantial difference to the effectiveness of the intervention in most studies. Two studies which reported improvements in prescribing due to A&F, the pharmacist was a colleague to the prescribers,^34,39^ but in another study which demonstrated a positive effect from A&F, the pharmacist was neither a colleague nor interprofessional collaborator.^36^

Four studies reported the contributing pharmacists’ years of experience^32,34,36,37^; the median was 16 (range 0^34^ to 30^32^) years since registration. Pharmacists undertook study-specific training in 7 studies, e.g. communication skills, evidence-based medicine methodology; increased appropriate prescribing in the intervention group was observed in 4 of these studies.^32,36,37,40^ In 5 studies, the pharmacist reviewed prescriptions and records for individual patients^34,35,37,39,40^ and presented feedback to individual participants; all 5 studies showed improved prescribing in their intervention groups.

## Discussion

The results of this review indicate that A&F interventions in general practice involving pharmacists tend to be effective at improving prescribing compared with no intervention or non-A&F interventions, such as education only or distribution of guidelines alone. The effect size of these pharmacist-related A&F interventions were moderate and were similar in magnitude to those reported in earlier reviews of A&F interventions delivered by different healthcare professionals.^24,46^ Furthermore, the findings indicate the effectiveness of the pharmacist-related A&F is associated with specific pharmacist characteristics, e.g. receipt of focussed training and intervention components, e.g. delivery of feedback concerning prescribing for individual patients.

### Comparison with existing literature

This review adds to existing evidence of the effectiveness of pharmacist involvement in interventions to improve prescribing in a range of healthcare settings.^17,47^ Recent systematic reviews^48,49^ reported that academic detailing delivered by pharmacists, both singly and as part of a multifaceted intervention, was effective in reducing adverse drug events and medication errors, respectively. In academic detailing, the educator is typically a health professional based outside the participant’s practice^50^; the professional may be a pharmacist.^51^ This current review included studies of multifaceted interventions, which included pharmacist-led education in addition to pharmacist conduct of prescribing audits and delivery of feedback. Pharmacists in this review included colleagues, interprofessional collaborators and external experts, but the existence of a pre-existing relationship with target prescribers was not associated with the effectiveness of A&F interventions. The results suggest that interventions where pharmacists provide ongoing feedback on individual prescribing decisions may be more effective than those in which their involvement is either fleeting or based on sessions in pre-existing collaborations of prescribers.

The results of the current review differed from previous findings^24^ which have found that feedback of general information plus tailored action plans are more effective than feedback of general information only. Reports containing individual patient-level data appeared to have greater impact on prescribing than those containing team- or clinician-level data, but given the small number of studies in this review it is not possible to detect statistically significant differences.

Previous reviews have identified other influential features relating to the process of feedback, including the provision of feedback to groups and individuals,^52,53^ repeated provision of feedback,^24,52^ the use of a range of media used to convey feedback,^53^ and the role of clinical decision support systems.^54^ This current review concurs with previous findings about the effectiveness of providing both verbal and written feedback,^34–37^ but was inconclusive about the impact of providing multiple episodes of feedback.^32,40^ Verbal feedback, whether in-person or by telephone, was more effective than other modes of feedback.^32,34–37,39,40^ The inclusion of computerized decision support at the point of prescribing also contributed to the effectiveness of interventions.^32,34,36,37,39^ We identified additional features of interventions which may contribute to the effectiveness of A&F, including the provision of feedback about prescribing for individual patients^34–37,39^ and study-specific skills training for the pharmacist delivering the intervention.^32,36,37^ In the light of the small number of studies in this review, and the level of heterogeneity amongst them, comparisons must be treated with caution.

### Implications for policy and research

This review demonstrated that A&F interventions involving pharmacists have a moderate positive effect on prescribing in general practice settings. Successful A&F interventions involved pharmacists in providing ongoing support to physicians about their prescribing for individual patients as well as scenarios in which pharmacists partnered physicians in local prescribing groups. It was not possible to identify the optimal working relationship between the pharmacist leading the A&F intervention and participants (i.e. colleague or external contact) from this review. Successful interventions may seek to increase a positive prescribing behaviour or reduce inappropriate prescribing; the direction of change, i.e. increased or decreased prescribing behaviour, does not appear to be a determining factor in an intervention’s success.

Although this review suggests that A&F interventions involving pharmacists who have undertaken study-specific training may have a more positive effect on prescribing, information relating to the content of the training and about the pharmacist’s general level of experience and expertise was limited. These are topics which warrant further enquiry.

### Strengths and limitations

This is the first review to focus specifically on A&F interventions involving pharmacists as key contributors to improve prescribing in general practice settings. A pre-defined study protocol is publicly available. All included studies were cluster-randomized trials which focussed on enhanced roles for pharmacists in general practice settings. The risk of bias in most domains was generally assessed as low.

Although this review adopted a robust search strategy recommended by the Cochrane Information Retrieval Methods group and followed the Cochrane EPOC methodology for duplicate data extraction and risk of bias assessments, screening for pharmacist-led A&F studies was limited to titles and abstracts (from the main Cochrane review) to identify eligible studies. As such, it may not have captured all relevant studies where pharmacists were not mentioned in either the title or abstract. An additional study was identified from examination of the full text of a study already identified for inclusion in the review.^31^

Studies included in this review reported pharmacist interventions in relatively affluent healthcare settings. Opportunities for pharmacists to influence prescribing in settings with fewer resources may be limited.

Owing to the lack of existing studies directly comparing A&F against A&F with pharmacist involvement, it was not possible within this review to estimate the relative effects of specifically pharmacist-led feedback. It would be difficult to produce a straightforward hierarchy of the “best” healthcare professionals to deliver A&F, as this would entail examination of the moderating effects of a range of factors, such as training, feedback type, professional role, and team relationships.

Meta-analysis was performed where appropriate, but the level of heterogeneity amongst included studies was high. Owing to the low number of studies included in the meta-analysis, it was not possible to assess publication bias.

## Conclusions

By undertaking a range of responsibilities to promote evidence-based prescribing and encourage the judicious use of medicines, pharmacists make an important contribution to improving patient outcomes in general practice. A&F may be particularly well-matched with pharmacists’ professional skills and expertise.

Further exploration is needed to optimize their involvement in the provision of A&F interventions. The extent to which pharmacists currently deliver A&F interventions in general practice is unknown but is being explored in the United Kingdom as part of this research programme. The content and focus of training in undergraduate curricula and during foundation years should also be investigated to determine whether pharmacists are equipped to deliver interventions of this type as part of their general practice responsibilities.

## Supplementary material

Supplementary material is available at *Family Practice* online.

cmac150_suppl_Supplementary_Material_S1

cmac150_suppl_Supplementary_Material_S2

cmac150_suppl_Supplementary_Material_S3

## Data Availability

Data are available on reasonable request.

## References

[1] Guenette L , Maheu A, Vanier MC, Dugré N, Rouleau L, Lalonde L. Pharmacists practising in family medicine groups: what are their activities and needs? J Clin Pharm Ther. 2020;45(1):105–114. 31436893 10.1111/jcpt.13035

[2] Haua R , Harrison J, Aspden T. Pharmacist integration into general practice in New Zealand. J Prim Health Care. 2019;11(2):159–169. 32171359 10.1071/HC18103

[3] Hazen ACM , de Bont AA, Boelman L, Zwart DLM, de Gier JJ, de Wit NJ, Bouvy ML. The degree of integration of non-dispensing pharmacists in primary care practice and the impact on health outcomes: a systematic review. Res Social Adm Pharm. 2018;14(3):228–240. 28506574 10.1016/j.sapharm.2017.04.014

[4] NHS England. General practice forward view 2016 [accessed 2022 Jan]. https://www.england.nhs.uk/publication/general-practice-forward-view-gpfv/.

[5] National Health Service. NHS long term plan 2019 [accessed 2022 Jan]. https://www.longtermplan.nhs.uk/publication/nhs-long-term-plan/.

[6] Royal Pharmaceutical Society Wales. Models of care for pharmacy within primary care clusters. Cardiff, 2015.

[7] Scottish Government. Primary care investment 2015 [accessed 2022 Jan]. https://news.gov.scot/news/primary-care-investment.

[8] Office for Health Improvement and Disparities. Public health profiles 2022 [accessed 2022 Jan]. https://fingertips.phe.org.uk.

[9] Stocks SJ , Kontopantelis E, Akbarov A, Rodgers S, Avery AJ, Ashcroft DM. Examining variations in prescribing safety in UK general practice: cross sectional study using the Clinical Practice Research Datalink. BMJ. 2015;351:h5501. 26537416 10.1136/bmj.h5501 PMC4632209

[10] Sinnige J , Braspenning JC, Schellevis FG, Hek K, Stirbu I, Westert GP, Korevaar JC. Inter-practice variation in polypharmacy prevalence amongst older patients in primary care. Pharmacoepidemiol Drug Saf. 2016;25(9):1033–1041. 27133740 10.1002/pds.4016

[11] Trueman P , Lowson K, Blighe A, et al. Evaluation of the scale, causes and costs of waste medicines. 2010.

[12] The Kings Fund. The rising cost of medicines to the NHS: what’s the story? 2018.

[13] Flodgren G , Hall AM, Goulding L, et al. Tools developed and disseminated by guideline producers to promote the uptake of their guidelines. Cochrane Database Syst Rev. 2016(8). 10.1002/14651858.CD010669.pub2 PMC10506131 27546228

[14] Eccles M , Grimshaw J, Walker A, Johnston M, Pitts N. Changing the behavior of healthcare professionals: the use of theory in promoting the uptake of research findings. J Clin Epidemiol. 2005;58(2):107–112. 15680740 10.1016/j.jclinepi.2004.09.002

[15] Grimshaw JM , Eccles MP, Lavis JN, et al. Knowledge translation of research findings. Implement Sci. 2012;7(1):50. 22651257 10.1186/1748-5908-7-50 PMC3462671

[16] Wingler MJB , Stover KR, Barber KE, et al. An evaluation of pharmacist-led interventions for inpatient HIV-related medication errors. J Pharm Technol. 2019. 10.1177/8755122519856728 PMC6726876 34752524

[17] Maaskant JM , Tio MA, van Hest RM, Vermeulen H, Geukers VGM. Medication audit and feedback by a clinical pharmacist decrease medication errors at the PICU: an interrupted time series analysis. Health Sci Rep. 2018;1(3):e23. 30623062 10.1002/hsr2.23 PMC6200092

[18] Langford B , Chan AJ, Brown K, et al. High-vs. low-intensity prospective audit and feedback on internal medicine wards and impact on antimicrobial use at a community hospital. Open Forum Infect Dis. 2017:S489–SS90.

[19] Williams R , Keers R, Gude WT, Jeffries M, Davies C, Brown B, Kontopantelis E, Avery AJ, Ashcroft DM, Peek N. SMASH! The Salford medication safety dashboard. J Innov Health Inform. 2018;25(3):183–193. 30398462 10.14236/jhi.v25i3.1015

[20] Wallis K , Tuckey R. Safer Prescribing and Care for the Elderly (SPACE): feasibility of audit and feedback plus practice mail-out to patients with high-risk prescribing. J Prim Health Care. 2017;9(2):145–152. 29530226 10.1071/HC17018

[21] Hunt L , Bohan J, McKie R, et al. Evaluation of an audit and feedback intervention to improve acute respiratory tract (ARI) antibiotic prescribing in outpatients. Open Forum Infect Dis. 2016;3.

[22] Johnson MJ , May CR. Promoting professional behaviour change in healthcare: what interventions work, and why? A theory-led overview of systematic reviews. BMJ Open. 2015;5(9):e008592. 10.1136/bmjopen-2015-008592 PMC4593167 26423853

[23] World Health Organization Europe. Using audit and feedback to health professionals to improve the quality and safety of health care. Copenhagen, 2010.

[24] Ivers N , Jamtvedt G, Flottorp S, et al. Audit and feedback: effects on professional practice and healthcare outcomes. Cochrane Database Syst Rev. 2012(6):CD000259. 22696318 10.1002/14651858.CD000259.pub3 PMC11338587

[25] Brehaut JC , Colquhoun HL, Eva KW, Carroll K, Sales A, Michie S, Ivers N, Grimshaw JM. Practice feedback interventions: 15 suggestions for optimizing effectiveness. Ann Intern Med. 2016;164(6):435–441. 26903136 10.7326/M15-2248

[26] Colquhoun H , Carroll K, Eva KW, et al. Advancing the literature on designing audit and feedback interventions: identifying theory-informed hypotheses. Implement Sci. 2017;12(1):117. 28962632 10.1186/s13012-017-0646-0 PMC5622490

[27] Moher D , Liberati A, Tetzlaff J, Altman DG; PRISMA Group. Preferred reporting items for systematic reviews and meta-analyses: the PRISMA statement. PLoS Med. 2009;6(7):e1000097. 19621072 10.1371/journal.pmed.1000097 PMC2707599

[28] Ivers N , Antony J, Konnyu K, et al. Audit and feedback: effects on professional practice [protocol for a Cochrane review update]. 2022. doi:10.5281/zenodo.6354035

[29] Higgins JPT , Altman DG, Gøtzsche PC, Jüni P, Moher D, Oxman AD, Savovic J, Schulz KF, Weeks L, Sterne JAC; Cochrane Bias Methods Group. The Cochrane Collaboration’s tool for assessing risk of bias in randomised trials. BMJ. 2011;343:d5928. 22008217 10.1136/bmj.d5928 PMC3196245

[30] Higgins JPT ; Cochrane Collaboration. Cochrane handbook for systematic reviews of interventions. 2nd ed. Hoboken (NJ): Wiley-Blackwell; 2020.

[31] Trietsch J , van Steenkiste B, Grol R, Winkens B, Ulenkate H, Metsemakers J, van der Weijden T. Effect of audit and feedback with peer review on general practitioners’ prescribing and test ordering performance: a cluster-randomized controlled trial. BMC Fam Pract. 2017;18(1):53. 28407754 10.1186/s12875-017-0605-5 PMC5390393

[32] Vervloet M , Meulepas MA, Cals JW, Eimers M, van der Hoek LS, van Dijk L. Reducing antibiotic prescriptions for respiratory tract infections in family practice: results of a cluster randomized controlled trial evaluating a multifaceted peer-group-based intervention. NPJ Prim Care Respir Med. 2016;26:15083. 26845640 10.1038/npjpcrm.2015.83 PMC4741286

[33] Magrini N , Formoso G, Capelli O, et al. Long term effectiveness on prescribing of two multifaceted educational interventions: results of two large scale randomized cluster trials. PLoS One. 2014;9(10):e109915. 25329386 10.1371/journal.pone.0109915 PMC4201458

[34] Avery AJ , Rodgers S, Cantrill JA, et al. A pharmacist-led information technology intervention for medication errors (PINCER): a multicentre, cluster randomised, controlled trial and cost-effectiveness analysis. Lancet. 2012;379(9823):1310–1319. 22357106 10.1016/S0140-6736(11)61817-5 PMC3328846

[35] Bregnhoj L , Thirstrup S, Kristensen MB, et al. Combined intervention programme reduces inappropriate prescribing in elderly patients exposed to polypharmacy in primary care. Eur J Clin Pharmacol. 2009;65(2):199–207. 18807252 10.1007/s00228-008-0558-7

[36] Fretheim A , Oxman AD, Havelsrud K, Treweek S, Kristoffersen DT, Bjørndal A. Rational prescribing in primary care (RaPP): a cluster randomized trial of a tailored intervention. PLoS Med. 2006;3(6):e134. 16737346 10.1371/journal.pmed.0030134 PMC1472695

[37] Clyne B , Smith SM, Hughes CM, Boland F, Bradley MC, Cooper JA, Fahey T; OPTI-SCRIPT study team. Effectiveness of a multifaceted intervention for potentially inappropriate prescribing in older patients in primary care: a cluster-randomized controlled trial (OPTI-SCRIPT study). Ann Fam Med. 2015;13(6):545–553. 26553894 10.1370/afm.1838 PMC4639380

[38] Crotty M , Whitehead C, Rowett D, Halbert J, Weller D, Finucane P, Esterman A. An outreach intervention to implement evidence based practice in residential care: a randomized controlled trial [ISRCTN67855475]. BMC Health Serv Res. 2004;4(1):6. 15066200 10.1186/1472-6963-4-6 PMC415557

[39] Pape GA , Hunt JS, Butler KL, Siemienczuk J, LeBlanc BH, Gillanders W, Rozenfeld Y, Bonin K. Team-based care approach to cholesterol management in diabetes mellitus: two-year cluster randomized controlled trial. Arch Intern Med. 2011;171(16):1480–1486. 21911633 10.1001/archinternmed.2011.417

[40] Lim WY , Singh A, Ng LM, et al. The impact of a prescription review and prescriber feedback system on prescribing practices in primary care clinics: a cluster randomised trial. BMC Fam Pract. 2018;19(1):120. 30025534 10.1186/s12875-018-0808-4 PMC6053727

[41] World Health Organization Guidelines Subcommittee. 1999 World Health Organization-International Society of Hypertension Guidelines for the Management of Hypertension. J Hypertens. 2020;17(2):151–183. 10067786

[42] British National Formulary. BNF publications 2020 [accessed 2022 Jan]. https://www.bnf.org/.

[43] Dutch College of General Practitioners. Nederlands Huisartsen Genootschap (NHG) 2019 [accessed 2022 Jan]. https://www.nhg.org/english/about-us.

[44] Rognstad S , Brekke M, Fetveit A, Spigset O, Wyller TB, Straand J. The Norwegian General Practice (NORGEP) criteria for assessing potentially inappropriate prescriptions to elderly patients. A modified Delphi study. Scand J Prim Health Care. 2009;27(3):153–159. 19462339 10.1080/02813430902992215 PMC3413187

[45] Sterne JAC , Sutton AJ, Ioannidis JPA, Terrin N, Jones DR, Lau J, Carpenter J, Rücker G, Harbord RM, Schmid CH, et al. Recommendations for examining and interpreting funnel plot asymmetry in meta-analyses of randomised controlled trials. BMJ. 2011;343:d4002. 21784880 10.1136/bmj.d4002

[46] Kroon D , Steutel NF, Vermeulen H, et al. Effectiveness of interventions aiming to reduce inappropriate drug prescribing: an overview of interventions. J Pharm Health Serv Res. 2021;12(3):423–433.

[47] Jeffries M , Keers RN, Phipps DL, et al. Developing a learning health system: insights from a qualitative process evaluation of a pharmacist-led electronic audit and feedback intervention to improve medication safety in primary care. PLoS One. 2018;13(10):e0205419. 30365508 10.1371/journal.pone.0205419 PMC6203246

[48] Ali S , Salahudeen MS, Bereznicki LRE, Curtain CM. Pharmacist-led interventions to reduce adverse drug events in older people living in residential aged care facilities: a systematic review. Br J Clin Pharmacol. 2021;87(10):3672–3689. 33880786 10.1111/bcp.14824

[49] Jaam M , Naseralallah LM, Hussain TA, Pawluk SA. Pharmacist-led educational interventions provided to healthcare providers to reduce medication errors: a systematic review and meta-analysis. PLoS One. 2021;16(6):e0253588. 34161388 10.1371/journal.pone.0253588 PMC8221459

[50] Soumerai SB , Avorn J. Principles of educational outreach (‘academic detailing’) to improve clinical decision making. JAMA 1990;263(4):549–456. 2104640

[51] Dreischulte T , Donnan P, Grant A, Hapca A, McCowan C, Guthrie B. Safer Prescribing—a trial of education, informatics, and financial incentives. N Engl J Med. 2016;374(11):1053–1064. 26981935 10.1056/NEJMsa1508955

[52] Foster M , Presseau J, McCleary N, Carroll K, McIntyre L, Hutton B, Brehaut J. Audit and feedback to improve laboratory test and transfusion ordering in critical care: a systematic review. Implement Sci. 2020;15(1):46. 32560666 10.1186/s13012-020-00981-5 PMC7303577

[53] Le Grand Rogers R , Narvaez Y, Venkatesh AK, Fleischman W, Hall MK, Taylor RA, Hersey D, Sette L, Melnick ER. Improving emergency physician performance using audit and feedback: a systematic review. Am J Emerg Med. 2015;33(10):1505–1514. 26296903 10.1016/j.ajem.2015.07.039

[54] Kwan JL , Lo L, Ferguson J, Goldberg H, Diaz-Martinez JP, Tomlinson G, Grimshaw JM, Shojania KG. Computerised clinical decision support systems and absolute improvements in care: meta-analysis of controlled clinical trials. BMJ. 2020;370:m3216. 32943437 10.1136/bmj.m3216 PMC7495041

