# Supplementary material for: Audit and feedback interventions involving pharmacists to influence prescribing behaviour in general practice: a systematic review and meta-analysis
Source: Fam Pract. 2023 Jan 12;40(5-6):615–28. doi: 10.1093/fampra/cmac150 (PMC10745261; doi:10.1093/fampra/cmac150)
Supplement: cmac150_suppl_Supplementary_Material_S1 [file cmac150_suppl_supplementary_material_s1.docx]

**SUPPLEMENTARY MATERIAL 1 – PRISMA-COMPLIANT ABSTRACT**

**Background**

Pharmacists, as experts in medicines, are increasingly employed in general medical practices. Audit and feedback (A&F) interventions are effective in achieving behaviour change, including prescribing. The extent to which pharmacists are involved in A&F interventions to influence prescribing in general practice is unknown.

**Objectives**

To assess the effectiveness of A&F interventions involving pharmacists as key contributors on prescribing in general practice compared with no A&F intervention or usual care. To describe the features of these A&F interventions and the pharmacists involved.

**Data sources**

Electronic databases were searched in 2012, 2019 and 2020 (MEDLINE, EMBASE, CINAHL, Cochrane Central Register of Controlled Trials, Science Citation Index, Social Sciences Citation Index, ISI Web of Science). Standard systematic review methods were applied to trial identification and selection.

**Study eligibility criteria, participants and interventions**

Randomised studies of interventions including A&F in general practice, which involved pharmacists as key contributors and healthcare professionals as participants.

**Study appraisal and synthesis methods**

The Cochrane Risk of Bias tools were applied. Results were summarised descriptively. Heterogeneity was assessed. A random-effects meta-analysis was conducted where studies were sufficiently homogenous in design and outcome.

**Results**

Eleven cluster-randomised studies from nine countries were included. Risk of bias across most domains was low. Interventions were mostly designed to reduce unsafe prescribing or the use of specific drugs or therapeutic categories. Meta-analysis of six studies showed improved prescribing outcomes (pooled risk ratio 0.78, 95% CI 0.64-0.94). Interventions which included both verbal and written feedback or computerised decision support for prescribers were more effective. Pharmacists who received study-specific training, provided ongoing support to prescribers or who reviewed prescribing for individual patients, contributed to more effective interventions.

**Limitations**

Substantial heterogeneity in study context, intervention components, and outcomes was observed across studies.

**Conclusions & implications of key findings**

A&F interventions involving pharmacists can lead to small improvements in evidence-based prescribing in general practice settings. Future implementation of A&F within general practice should compare different ways of involving pharmacists to determine how to optimise effectiveness.

**Systematic review registration number**

PROSPERO registration number CRD42020194355
