# Supplementary material for: Audit and feedback interventions involving pharmacists to influence prescribing behaviour in general practice: a systematic review and meta-analysis
Source: Fam Pract. 2023 Jan 12;40(5-6):615–28. doi: 10.1093/fampra/cmac150 (PMC10745261; doi:10.1093/fampra/cmac150)
Supplement: cmac150_suppl_Supplementary_Material_S3 [file cmac150_suppl_supplementary_material_s3.docx]

**SUPPLEMENTARY MATERIAL 3 – A&F REVIEW UPDATE SEARCH STRATEGY (DRAFT) & INFORMATION SCIENTIST SEARCH NOTES**

Revised search strategy for MEDLINE developed by **Paul Miller**

| Ovid MEDLINE(R) In-Process & Other Non-Indexed Citations and Ovid MEDLINE(R) 1946 to Present |
| --- |

| **#** | **Searches** | **Results** |
| --- | --- | --- |
| 1 | (audit* adj3 (feedback or fed back)).ti,ab. | 2137 |
| 2 | clinical audit/ | 984 |
| 3 | medical audit/ | 15654 |
| 4 | nursing audit/ | 2980 |
| 5 | dental audit/ | 389 |
| 6 | management audit/ | 2426 |
| 7 | benchmarking/ | 11008 |
| 8 | "commission on professional and hospital activities"/ | 238 |
| 9 | feedback/ | 27107 |
| 10 | feedback, psychological/ | 2637 |
| 11 | utilization review/ | 7421 |
| 12 | drug utilization review/ | 3334 |
| 13 | concurrent review/ | 379 |
| 14 | peer review, health care/ | 1349 |
| 15 | (audit or audits or auditing).ti,ab. | 29083 |
| 16 | (feedback or fed back).ti,ab. | 96335 |
| 17 | (review adj4 record?).ti,ab. | 15493 |
| 18 | chart review?.ti,ab. | 27401 |
| 19 | (practice data or hospital* data).ti,ab. | 4056 |
| 20 | benchmark*.ti,ab. | 22775 |
| 21 | (scorecard? or score card? or reportcard? or report card?).ti,ab. | 1988 |
| 22 | (dashboard* or dash board*).ti,ab. | 487 |
| 23 | patient registry.ti,ab. | 1137 |
| 24 | (business intelligence or process control).ti,ab. | 2111 |
| 25 | (panel adj (management or support or view*)).ti,ab. | 51 |
| 26 | (performance adj2 (scor* or measur* or monitor* or improv*)).ti,ab. | 57953 |
| 27 | or/2-26 | 290546 |
| 28 | exp health personnel/ | 398550 |
| 29 | (health* professional? or health care professional? or health* team? or health care team? or physician? or clinician? or nurs* or provider* or doctor? or intern? or caregiver? or gp? or therapist? or health* personnel or health care personnel or practitioner? or resident? or pharmacist?).ti,ab. | 1241955 |
| 30 | quality assurance, health care/ | 51586 |
| 31 | quality indicators, health care/ | 11749 |
| 32 | quality improvement/ | 9440 |
| 33 | quality of health care/ | 61621 |
| 34 | (quality adj (assurance or improvement or control or indicator?)).ti,ab. | 73140 |
| 35 | (health care quality or healthcare quality or quality of healthcare or quality of health care or quality of care).ti,ab. | 41317 |
| 36 | ((influenc* or chang*) adj3 (behaviour* or behavior* or practice)).ti,ab. | 71172 |
| 37 | or/28-36 | 1625936 |
| 38 | exp randomized controlled trial/ | 418041 |
| 39 | controlled clinical trial.pt. | 92294 |
| 40 | randomi#ed.ti,ab. | 438602 |
| 41 | randomly.ti,ab. | 246498 |
| 42 | random allocation/ | 87117 |
| 43 | clinical trials as topic.sh. | 180211 |
| 44 | trial.ti. | 150297 |
| 45 | or/38-44 | 1057109 |
| 46 | exp animals/ not humans/ | 4156219 |
| 47 | 45 not 46 | 957189 |
| 48 | 1 or (27 and 37) | 68998 |
| 49 | 47 and 48 | 5925 |
| 50 | (2010* or 2011* or 2012* or 2013* or 2014* or 2015*).ed,ep,yr. | 6702170 |
| 51 | 49 and 50 | 2785 |

Audit & feedback search notes

- Tested on OVID medline
- Tested each step using 83 included studies from current review (ones that I had the UI numbers for already):
  - (8129501 or 14695072 or 12879828 or 10166596 or 9431333 or 9495403 or 2383413 or 10600428 or 10551192 or 3230459 or 2066823 or 8347386 or 3735627 or 7078287 or 11356439 or 6679239 or 9949744 or 12837711 or 12792007 or 12358871 or 6700282 or 14660997 or 9388790 or 10942103 or 11242721 or 11280691 or 1511147 or 9700115 or 3520177 or 10506237 or 9807540 or 2218150 or 10478162 or 10673488 or 11401608 or 10625023 or 9734786 or 12632705 or 10980076 or 7392153 or 8947715 or 2013952 or 10645510 or 7432374 or 3888133 or 9663162 or 3092976 or 9233326 or 6891147 or 11387182 or 11294376 or 4045397 or 10024260 or 3892184 or 12668540 or 15065730 or 11232071 or 11490051 or 12014939 or 7822993 or 10555719 or 10533949 or 11022366 or 14521638 or 7837822 or 6811994 or 12236277 or 7617573 or 9565408 or 6513620 or 9582043 or 12012145 or 3736141 or 10847872 or 10508816 or 11743151 or 14516301 or 6738143 or 7861879 or 3807449 or 10533946).ui.
- Changed RCT filter to (modified) Cochrane RCT filter - still misses 5 of 83 includes, better than 7 of 83 for previous RCT filter: <http://handbook.cochrane.org/chapter_6/box_6.4.d_cochrane_hsss_2008_sensprec_ovid.htm>
- Added “fed back” to natural language terms
- Added majority of terms suggested by Ben
- Looked through a sample of RCTs retrieved by this line and RCT filter; nothing relevant found so not added to search: ((pathway or ICP or guideline or CPG) adj3 (analy* or visuali*)).tw.
- One included study not found using .tw. but found using .ti,ab. – altered all natural language terms to .ti,ab.
- Tested MeSH term quality indicators, health care/ - added as part of the set that limits retrieval to quality
- Natural language for health personnel/ heading is restricted in original by adjacency to (skill or skills or behaviour or behavior or competence). Removed restriction and expanded terms as this is major cause of missed papers
- Removed irrelevant MeSH from set that limits retrieval to quality
- Moved “performance” to intervention terms, retrieval almost doubled so restricted performance using adjacency to (scor* or measur* or monitor* or improv*)
- Changed (review adj3 record?) to (review adj4 record?) to pick up one more included study from the 83
- 77 of 83 includes retrieved. Of 6 not retrieved, 5 are excluded by the study design filter.
- Overall retrieval increased by approx. 1200 on medline
